# Clinical Efficacy and Safety of Eight Traditional Chinese Medicine Combined with Entecavir in the Treatment of Chronic Hepatitis B Liver Fibrosis in Adults: A Network Meta-Analysis

**DOI:** 10.1155/2020/7603410

**Published:** 2020-09-30

**Authors:** Tao Wang, Wei Jin, Qianqian Huang, Haotian Li, Yun Zhu, Honghong Liu, Huadan Cai, Jiabo Wang, Ruilin Wang, Xiaohe Xiao, Yanling Zhao, Wenjun Zou

**Affiliations:** ^1^College of Pharmacy, Chengdu University of Traditional Chinese Medicine, Chengdu 611137, China; ^2^Department of Pharmacy, 302 Military Hospital of China, Beijing 100039, China; ^3^Hospital of Chengdu University of Traditional Chinese Medicine, Chengdu 610075, China; ^4^Integrative Medical Center, 302 Military Hospital of China, Beijing 100039, China; ^5^China Military Institute of Chinese Medicine, 302 Military Hospital of China, Beijing 100039, China

## Abstract

**Background:**

Traditional Chinese medicine (TCM) is used as an adjuvant drug for the treatment of chronic hepatitis B liver fibrosis and is used frequently. We still do not know which TCM has the best curative effect as an adjuvant drug. Therefore, we decided to use network meta-analysis to solve this problem.

**Methods:**

We used the RevMan software (5.3) and Stata software (13.0) to achieve this network meta-analysis (NMA). The primary outcomes of this study were HA, LN, PCIII, and IV-C; the secondary outcomes of this study were AST, ALT, and HBV-DNA negative conversion rate, and the Cochrane risk-of-bias tool was used to assess the quality of the included studies. For all outcomes, the scissors funnel plot, Egger test, and Begg test were used to detect publication bias, and sensitivity analysis was used to investigate the stability of the results. And the meta-regression was used to explore the source of heterogeneity.

**Results:**

A total of 34 articles were included in this study. The study involved a total of 3199 patients, of which 1578 were assigned to the control group and 1621 patients were assigned to the experimental group. The number of men and women is roughly equal, and the average age is about 43 years old. In addition, nine treatment strategies were involved in this study. The combination of TCM and entecavir can significantly improve the patients' HA, LN, PCIII, IV-C, AST, ALT, and HBV-DNA negative conversion rates. The comprehensive evaluation results showed that FHC combined with entecavir has more advantages than other treatment strategies.

**Conclusion:**

For improving the HBV-DNA negative conversion rates, adding TCM to the therapeutic strategies does not seem to show absolute superiority. Finally, FHC combined with entecavir is the best therapeutic strategy.

## 1. Introduction

Chronic hepatitis B (CHB) is one of the most serious infectious diseases in the world today. It has extremely high morbidity and mortality, and it is a serious threat to human health [1, 2]. Every year, more than 600,000 people die from hepatitis B virus (HBV)-related diseases in the world [3]. Liver fibrosis, which as a process of hepatic repair and healing, can eventually develop into cirrhosis or even liver cancer if the liver damage factors cannot be removed over a long period of time. In addition, hepatitis B virus is one of the most common causes of liver cirrhosis in China. Statistical studies have shown that 3% of patients with chronic hepatitis B (CHB) have decompensated liver cirrhosis every year, 2% to 8% of patients have primary liver cancer, and once they progress to decompensation liver cirrhosis, its 5-year cumulative survival rate was only 14% to 35% [4]. Therefore, for liver fibrosis, which as the starting point for serious liver disease, if this process can be well inhibited, it will be able to prevent liver disease from worsening in time.

At present, nucleoside (acid) analogues are mainly used clinically to treat hepatitis B liver fibrosis, such as adefovir dipivoxil, entecavir, and tenofovir disoproxil, which mainly through the continuous suppression of HBV-DNA replication to alleviate B hepatitis liver fibrosis. In particular, entecavir has become one of the main first-line drugs for the treatment of liver fibrosis in chronic hepatitis B in China. However, the use of Western medicine (such as entecavir) for the treatment of chronic hepatitis B liver fibrosis has a long cycle of treatment and even requires the patient to take medicine for life, causing a certain burden on the patient's family and greatly affecting the patient's own quality of life. And entecavir is a kind of guanine nuclear analog, although it has good safety, but there are still some studies reported that it caused lactic acidosis [5]. However, the cost of TCM treatment is relatively low, and TCM can act on multiple targets in the body at the same time. Many studies have shown that the use of Chinese and Western medicine in the treatment of chronic hepatitis B liver fibrosis can achieve better effect [6].

In China, there are many types of TCM used by clinicians in the treatment of chronic hepatitis B liver fibrosis. Among them, there are 8 kinds of TCM used as first-line drugs and included in the Chinese Pharmacopoeia: Anluo Huaxian Pill (AHP), Dahuang Zhechong Capsule (DZC), and Tanshinone Capsule (TSC), Danshen Injection (DI), Fuzheng Huayu Capsule (FHC), Biejia Ruangan Tablet (BRT), Danshendi Tablet (DDT), and Liuwei Wuling Tablet (LWT). The efficacy of these commonly used TCM is definite, and combined with the current first-line drug entecavir can significantly improve the clinical efficacy. This conclusion is not only due to long-term clinical experience. In recent years, many scholars have used meta-analysis and systematic review of evidence-based medical research methods to analyze the efficacy of the joint use of Chinese and Western medicine. Wang et al. showed that FHC combined with entecavir can significantly improve liver function and liver fibrosis in chronic hepatitis B patients [7]. Duan et al. also reached a similar conclusion when studying the effect of BRT combined with entecavir on the improvement of chronic hepatitis B cirrhosis [8]. In addition, LWT and AHP are also good auxiliary drugs [9, 10]. Although some researchers use meta-analysis methods to compare different treatment strategies, each comparison can only solve the problem of which of the two treatment strategies is better. When doctors choose treatment strategies, there are many kinds of TCM that can be selected, and which kind of TCM can achieve the best effect when used as an auxiliary medicine. This is still a problem that cannot be ignored. Therefore, we use the NMA to compare and rank the safety and effectiveness of the 8 TCMs which often used for adjuvant treatment of chronic hepatitis B liver fibrosis.

## 2. Materials and Methods

### 2.1. Search Strategy and Selection Criteria

We searched China National Knowledge Infrastructure, Wanfang Database, VIP medicine information system, PubMed, Embase, and Cochrane Library. The search time ranges from database establishment to May 2018. The initial search items were used as follows: “Fuzheng Huayu capsule,” “Biejia Ruangan Tablets,” “Liuwei Wuling Tablets,” “Anluo Huaxian Pills,” “Dahuang Zhechong Pills,” “Tanshinone capsules,” “Danshen Injection,” “Entecavir,” and “Chinese Medicine” [Title/Abstract] AND “Chronic hepatitis B liver fibrosis” [Title/Abstract] OR “Chronic hepatitis B” [Title/Abstract] OR “Liver Fibrosis” [Title/Abstract] OR “Hepatitis B” [Title/Abstract]. And the full text of the search results is downloaded. The inclusion criteria were as follows: (1) all randomized controlled trials (RCTs) and semirandomized controlled trials of Chinese Medicine combine with entecavir were included. (2) Chronic hepatitis B liver fibrosis was diagnosed according to definite diagnostic criteria. (3) The studies with an experimental group using entecavir combined with Chinese medicine and the control using entecavir were included. (4) The gender and age of patients were not limited. (5) The language of the literature is not limited. The exclusion criteria were as follows: (1) the repeated published literature, (2) studies with incomplete or incorrect data, (3) control group combined with other medicine during treatment, and (4) animal experiments and the review literature. And all patients were not treated with drugs such as interferon or nucleoside (acid) for six months before treatment.

### 2.2. Data Extraction and Quality Assessment

Three researchers (Tao Wang, Yanling Zhao, and Haotian Li) independently performed data extraction and quality assessment. Two researchers (Qianqian Huang and Huadan Cai) formed a review team to independently verify the accuracy of data extraction, quality assessment, and all materials for this study. Basic information was extracted from the included studies (name of the study included, average age of patients, number of patients, therapeutic strategies, mode of administration, dose administered, course of treatment, outcome, etc.) (Supplementary Table 1). The primary outcomes of this study were HA, LN, PCIII, and IV-C; the secondary outcomes of this study were AST, ALT, and HBV-DNA negative conversion rate, and the Cochrane Risk of Bias tool was used to assess the quality of the included studies. All the differences that occurred in the study were discussed by three researchers, and they were then agreed by the review team.

### 2.3. Statistical Analysis

We performed this study using RevMan software (5.3) and Stata software (13.0) and constructed a treatment strategy network. For the continuous variables, we calculated the normalized mean difference (MD), and for the dichotomous variable, we calculated the odds ratios (ORs). All of them were expressed with 95% CI. We compared each therapeutic strategy in pairs. Subsequently, *I*^2^ and chi-square tests were used to assess the heterogeneity between therapeutic strategies, and we use meta-regression to explore the source of heterogeneity. For all outcomes, the shear funnel plot, Egger test, and Begg test were used to detect publication bias, and sensitivity analysis was used to investigate the stability of the results. For all outcomes, the scissors funnel plot, Egger test, and Begg test were used to detect publication bias, and sensitivity analysis was used to investigate the stability of the results. Finally, we used the surface under the cumulative ranking curve (SUCRA) to rank the efficacy of the therapeutic strategy in each outcome. In addition, we did this NMA within a frequentist framework.

### 2.4. Statement

This study is registered with PROSPERO, number CRD42018095445. Finally, we need to state that the research funders did not interfere with any aspect of research design, data collection, data analysis, data interpretation, and article writing.

## 3. Results and Discussion

### 3.1. Results

#### 3.1.1. Quality and Characteristics of the Included Studies

**Figure 1:**
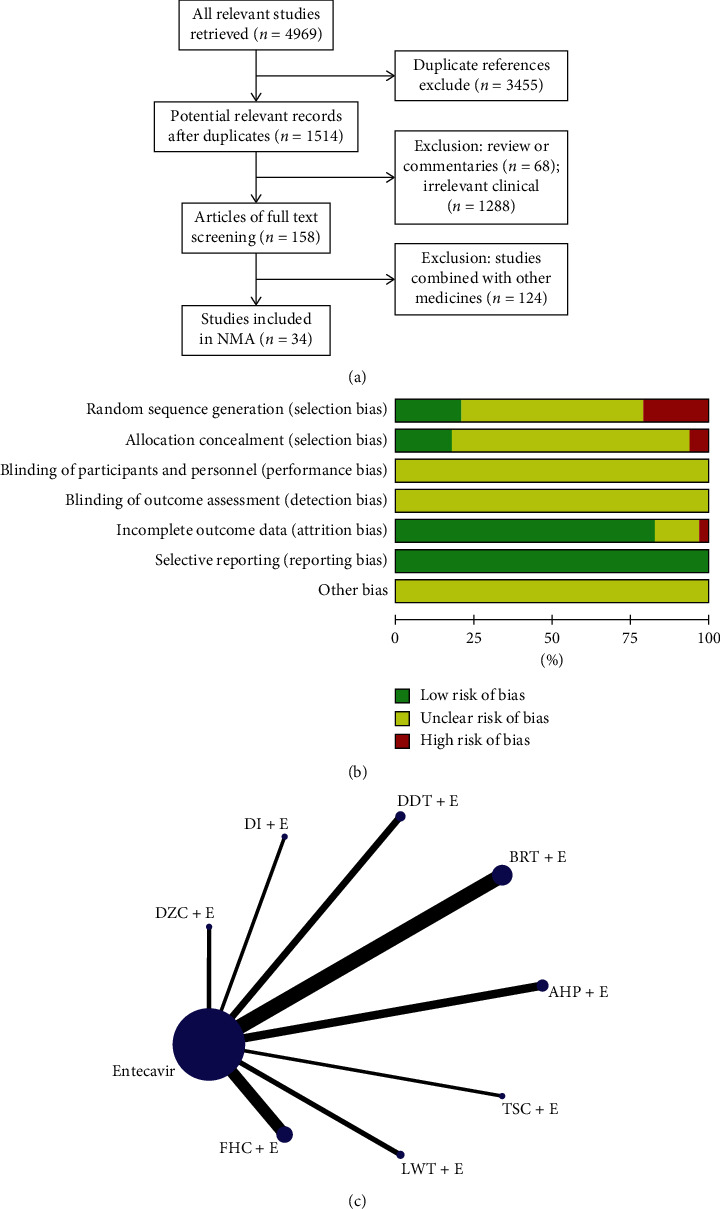
(a) Flowchart of study selection; (b) methodological quality assessment of the risk of bias for each included study; (c) network of eligible comparisons of efficacy of treatment.

Through the key words in the search strategy, a total of 4969 articles were obtained, of which 3455 were excluded from repetitive reports, 68 articles were excluded from the review, and 1288 articles that did not use TCM + entecavir as a treatment strategy were excluded. In addition, we also excluded 124 articles which the control group did not use entecavir alone or experiment group combination with a variety of TCM, and eventually included 34 articles [11–44] (Figure 1(a)). In the literature included in this study, a total of 7 studies reported the randomization method, 7 studies did not adopt a completely random grouping method, and the rest of the studies only mentioned randomized grouping but did not explicitly report the randomization method. In terms of data integrity, 28 studies have good data integrity, 5 studies cannot clearly determine whether the data are complete, and 1 study has missing data. In addition, unfortunately, all the included studies did not explicitly mention the blinding method, so they could not judge whether they had adopted the blinding method in the trial. However, there are no selective reports in all the included studies. We assessed the quality of included studies in accordance with the Cochrane risk-of-bias tool. Each evaluation principle was divided into “high risk,” “low risk,” and “unclear” (Figure 1(b)). All the studies included in this study were designed in parallel, and the test sites were all in China. All patients were diagnosed with chronic hepatitis B liver fibrosis according to definite diagnostic criteria and confirmed in regular hospitals. The control group was given entecavir, and the experimental group was combined with a traditional Chinese medicine based on the treatment strategy of the control group. The study involved a total of 3199 patients, of which 1578 were assigned to the control group and 1621 patients were assigned to the experimental group. The number of men and women is roughly equal, and the average age is about 43 years old. In addition, nine treatment strategies were involved in this study (Figure 1(c)).

#### 3.1.2. Primary Outcomes

**Figure 2:**
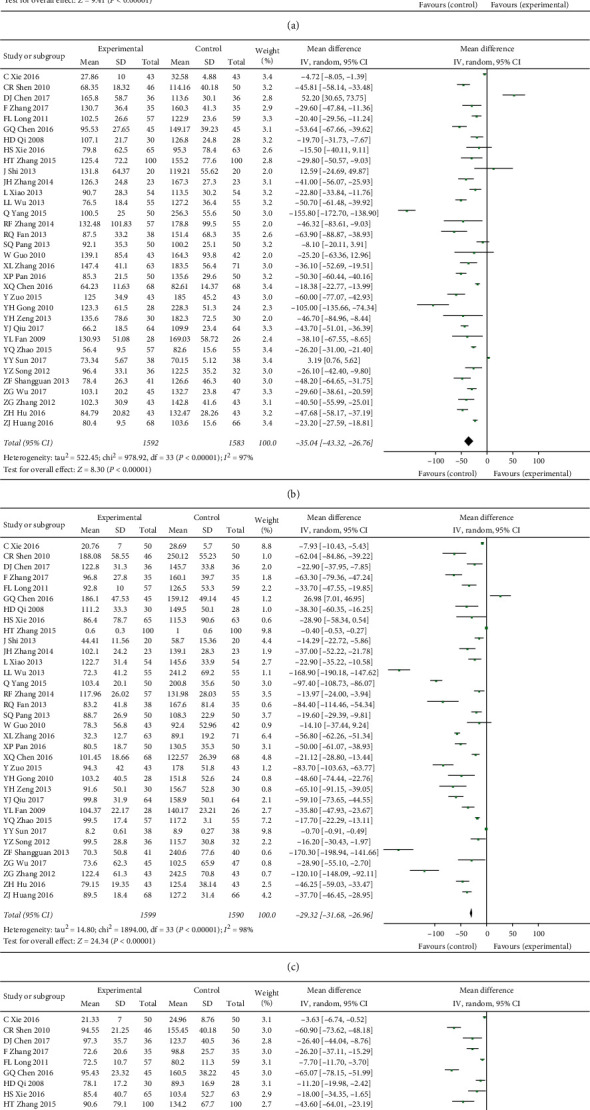
(a) The HA of TCM plus entecavir versus entecavir. (b) The LN of TCM plus entecavir versus entecavir. (c) The PCIII of TCM plus entecavir versus entecavir. (d) The IV-C of TCM plus entecavir versus entecavir. *I*^2^ and *P* are the criterion for the heterogeneity test, ◆ pooled odds ratio, —■— odds ratio, and 95% CI.

The primary outcomes of our study were liver fibrosis (HA, LN, PCIII, and IV-C). All the included studies have reported on these four indicators. Compared with entecavir alone, we found that TCM combined with entecavir can significantly improve liver fibrosis in patients (HA: MD = −57.15, 95% CI: −69.06−45.25, *P* < 0.00001; LN: MD = −35.04, 95% CI: −43.32−26.76, *P* < 0.00001; PCIII: MD = −29.32, 95% CI: −31.68−26.96, *P* < 0.00001; IV-C: MD = −38.98, 95% CI: −48.32−29.64, *P* < 0.00001) (Figure 2). We considered the internal heterogeneity of the studies, so we decided to use the random-effects model for data analysis. Moreover, in order to further verify the reliability of the results of this study, we use sensitivity analysis (Table 1) to explore the stability of the results and use funnel charts and statistical tests (Egger test and Begg test) to explore whether there is publication bias (Table 1 and Supplementary Figures 1–4). As a result, it was found that some of the results of this study were unstable, but most of the studies were stable and there was no significant publication bias. In addition, our meta-regression showed that the difference in sample size between the studies did not cause significant heterogeneity. The difference in therapeutic strategies is the main source of heterogeneity in this study (Table 1).

**Figure 3:**
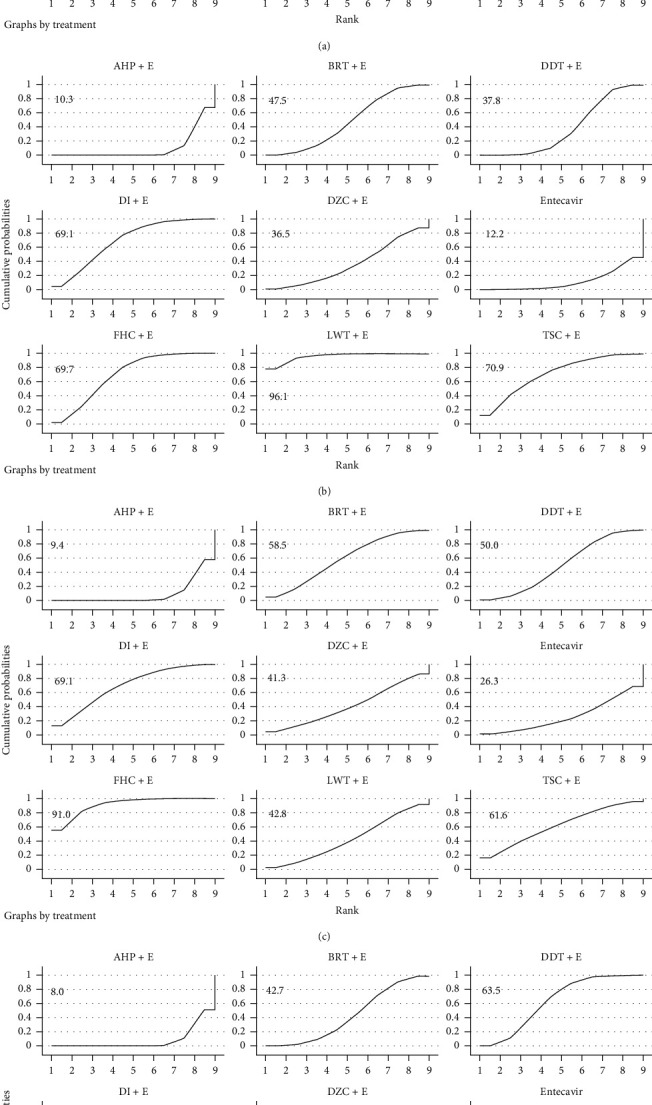
(a) Ranking for efficacy of HA; (b) ranking for efficacy of LN; (c) ranking for efficacy of PCIII; (d) ranking for efficacy of IV-C.

Based on the above results, we use Stata software to make a mixed comparison of all therapeutic strategies and calculate the OR value (Supplementary Tables 2–5), which also helps to eliminate the heterogeneity caused by the variety of therapeutic strategies between studies. Subsequently, based on the effect of various therapeutic strategies on liver fibrosis improvement, we use SUCAR charts to rank treatment decisions and calculate the average ranking of each therapeutic strategies to further explore which TCM as an adjuvant drug is the best therapeutic strategies (Figure 3). The results showed that FHC (2.25), TSC (3.25), and LWT (3.75) were the top three, followed by BRT (4.00), DI (4.50), DDT (4.75), DZC (6.00), and entecavir (7.50). The combination of AHP (9) and entecavir was the most unfavorable treatment strategy. Since direct and indirect comparisons between treatment strategies all contribute to the final results, we finally calculated the contribution of the direct and indirect comparisons to the final results in order to make the results of this study clearer, and the detailed results of this section are listed in the annex (Supplementary Figure 5).

#### 3.1.3. Secondary Outcomes

**Figure 4:**
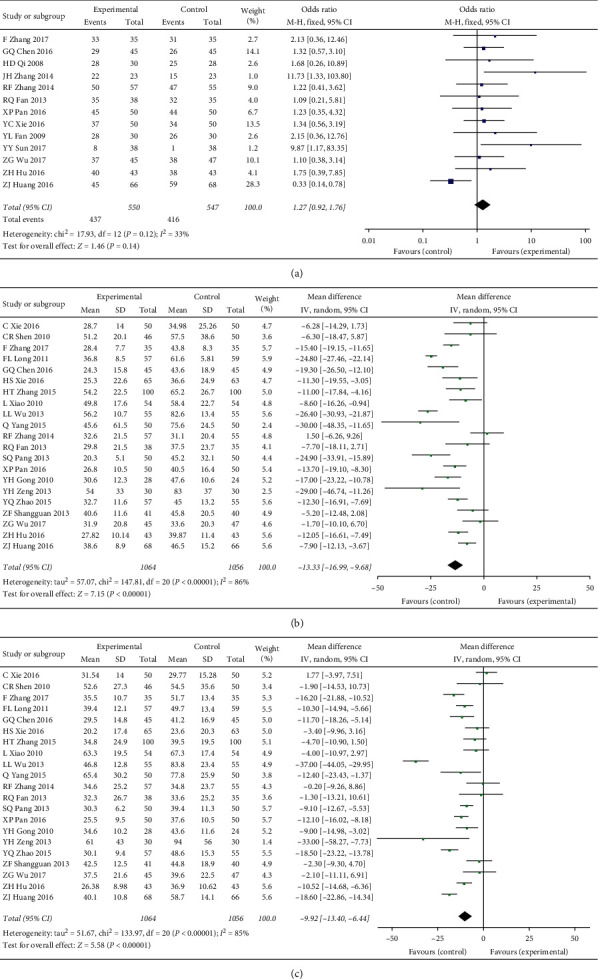
(a) The HBV-DNA negative conversion rate of TCM plus entecavir versus entecavir. (b) The ALT of TCM plus entecavir versus entecavir. (c) The AST of TCM plus entecavir versus entecavir. *I*^2^ and *P* are the criterion for the heterogeneity test, ◆ pooled odds ratio, —■— odds ratio, and 95% CI.

**Figure 5:**
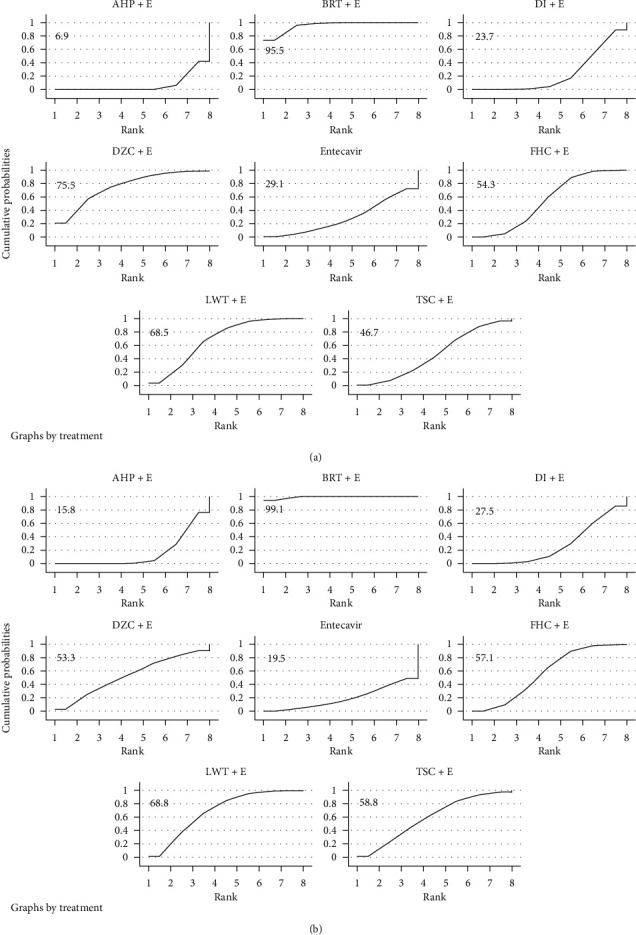
(a) Ranking for efficacy of ALT; (b) ranking for efficacy of AST.

There are 3 secondary outcomes in our study: liver function index (AST and ALT) and HBV-DNA negative conversion rate. A total of 13 studies reported the improvement of HBV-DNA negative conversion rate by TCM; 21 studies reported the improvement of ALT and AST by TCM. Our research found that (Figure 4) entecavir combined with TCM compared to entecavir alone and HBV-DNA negative conversion rate did not show a significant difference (OR = 1.27, 95% CI: 0.92–1.76, *P* = 0.14) but significantly improved ALT and AST (MD = −13.33, 95% CI: −16.99−9.68, *P* < 0.00001; MD = −9.92, 95% CI: −13.40−6.44, *P* < 0.00001). In order to further explore the stability of the results and whether the results were affected by potential biases, we performed sensitivity analyses (Table 1), scissors funnel plot (Supplementary Figures 6–8), Egger test, and Begg test (Table 1). We found that the results of this study were stable, there was a slight publication bias in HBV-DNA negative conversion rate, and there was no significant publication bias in liver function indicators. We then compared and ranked all of the treatment strategies (Supplementary Tables 6 and 7, Figure 5) and obtained the average ranking of the various treatment strategies. We found that BRT (1), LWT (2.5), DZC (3.5), and FHC (4) were more effective as adjuvant drugs in improving liver function, but AHP (8) achieved the worst results.

#### 3.1.4. Safety Assessment

A total of nine studies reported adverse events. These adverse reactions are mainly gastrointestinal discomfort such as nausea, vomiting, mild diarrhea, and loss of appetite; occasional allergies; and dizziness. However, none of the above symptoms were serious and disappeared after drug withdrawal. No related treatment was performed. Compared with the control group, the use of TCM did not have a significant impact on the incidence of adverse events. This shows that entecavir combined with TCM is safe in clinical use. In addition, details of the adverse events reported by the nine institutes are listed in the annex (Supplementary Table 8).

### 3.2. Discussion

Liver fibrosis caused by hepatitis B hepatitis is a reversible wound repair response characterized by the accumulation of extracellular matrix, which is the formation of scar tissue, which occurs after chronic or non-self-limiting liver disease, and it will eventually lead to cirrhosis. [45] In the world, the first-line drugs currently used are nucleoside (acid) analogues and interferon drugs, and their treatment cycles are generally long. In recent years, more and more research studies have found that TCM contains multiple active ingredients that can relieve or even reverse hepatitis B liver fibrosis. For example, *Salvia miltiorrhiza* and *Cordyceps sinensis*, they all have a variety of antifibrotic components, and in clinical use, there are many examples of using them to treat chronic hepatitis B liver fibrosis [46–48]. Rui's research further pointed out the pharmacological mechanisms of *Salvia miltiorrhiza* against liver fibrosis. The results of the study showed that *Salvia miltiorrhiza* water extract can inhibit the expression of GST-P and *α*-SMA during the process of hepatic fibrosis, and the alanine transaminase, aspartate aminotransferase, *γ*-glutamyltransferase, alkaline phosphatase, hyaluronic acid, direct bilirubin, and total bilirubin were significantly inhibited [49]. In addition, Peng et al. found that the main active components of *Cordyceps sinensis* against liver fibrosis are cordyceps polysaccharide, cordycepic acid, cordycepin, and ergosterol [50]. All these indicate that TCM is likely to play its unique role in the treatment of chronic hepatitis B liver fibrosis. Moreover, in long-term clinical practice, Chinese physicians have used and summarized many effective TCMs which are often combined with the current first-line drugs for the treatment of chronic hepatitis B liver fibrosis, and they were included in the Chinese Pharmacopoeia.

In this NMA, we found that 8 kinds of TCM as adjuvant medicines can improve liver fibrosis in patients with chronic hepatitis B liver fibrosis. The combination of FHC and entecavir is the best therapeutic strategy. It not only has a significant effect in improving the state of liver fibrosis in patients but also has a good effect in the improvement of liver function in patients. FZHY capsule is a commonly used TCM formula against liver fibrosis in clinic. It has been verified in research to exert antifibrosis effect through several mechanisms such as antilipid peroxidation and endothelial injury, inhibiting hepatic stellate cell activation, promoting matrix metalloproteinase activity, and degradation of pathological deposition of collagen. [51] In addition, there is an article using transcriptional profiling and miRNA-target network analysis to identify potential biomarkers for efficacy evaluation of FZHY formula-treated hepatitis B caused liver cirrhosis. [52] The use of AHP as an adjuvant drug in this study has shown a weaker effect and is lower than the single use of entecavir in the ranking, which is a very surprising result. In addition, the comprehensive evaluation results showed that the addition of TCM did not appear to have a significant impact on the improvement of HBV-DNA negative conversion rates, but when the therapeutic strategies were compared individually, some of the therapeutic strategies showed a significant improvement in the negative conversion rates, which is worth in-depth explore the phenomenon. First, we considered whether the results of this study were affected by publication bias. For this purpose, we conducted relevant statistical tests and scissors funnel plot to determine whether there was publication bias. However, the results showed that there were no significant publication biases other than HBV-DNA negative conversion rates in the seven outcomes of this study. The inconsistency in our study of HBV-DNA negative conversion rates is likely due to publication bias in this outcome measure. Therefore, this has also weakened the strength of the evidence in this NMA, which in judging whether the combined use of Chinese and Western medicine can enhance the improvement of HBV-DNA negative conversion rates. Subsequently, in order to evaluate the stability of the results of this network meta-analysis, we performed a dual modeling analysis of the fixed-effects model and the random-effects model for all indicators in turn. As a result, it was found that only the LN and IV-C outcomes were slightly unstable in all indicators, and there was no contradictory research result, and the remaining outcomes all had good stability. This NMA includes many documents, there are differences in the size of the sample and there are many types of treatment strategies, so in the process of research, we also pay attention to the heterogeneity between the studies. We not only used literature analysis methods to explore the sources of heterogeneity but also used meta-regression analysis to conduct relevant research. As mentioned earlier, in evidence-based medicine research, there are often small sample sizes of studies, and these small sample studies often show relatively unstable results. Therefore, we first examine whether it caused the heterogeneity between studies by using the sample size as a variable. It was found that when we used the sample size as a variable for regression analysis, the Tau2 of HBV-DNA negative conversion rates was 0, so the difference in sample size was the source of the heterogeneity of the outcome. Therefore, this also reduces our ability to demonstrate whether TCM as an auxiliary drug can increase the HBV-DNA negative conversion rates in another aspect. However, it is worth noting that the heterogeneity of the remaining indicators does not originate from the sample size. Subsequently, we considered whether the heterogeneity between studies was caused by the large number of treatment decisions, and the results were as we suspected. Meta-regression analysis showed that this factor actually contributed 71.64% and 80.32% of the heterogeneity of HA and LN. In addition, the gender ratio of patients in this study is balanced, but the age difference among patients in some studies is likely to be one of the reasons for heterogeneity. However, because some studies did not provide detailed reports on the age of patients, we did not consider this factor as a variable for meta-regression analysis. In terms of drug safety, there was no serious adverse reaction after the use of TCM combined with entecavir, and the combined use of drugs did not increase the risk of adverse events. Therefore, the use of 8 kinds of TCM combined with entecavir is safe.

Although there are slight instability and bias in the results of this study and there are limitations in the quality of RCTs, these have reduced the strength of the NMA. However, overall, this NMA still provides clinicians with detailed comparisons of common therapeutic strategies and provides reference for clinical use. In addition, in the future, large-scale, high-quality RCTs should be conducted to further provide evidence that FHC has efficacy and safety as adjuvant drugs for the treatment of hepatitis B and liver fibrosis in clinical applications.

## 4. Conclusions

The NMA showed that compared with entecavir, combined TCM did not increase the risk of adverse reactions and could significantly improve the effect of chronic hepatitis B liver fibrosis. In addition, in a variety of therapeutic strategies, the combination of FHC and entecavir is the best therapeutic strategy. However, TCM as a complementary drug has not shown significant advantages in improving the HBV-DNA negative conversion rates. Finally, due to the low quality of the included studies, the strength of the research results is limited. Therefore, strict RCTs are still needed in the future to further validate the effectiveness and safety of FHC as an adjuvant drug for the treatment of chronic hepatitis B liver fibrosis.

## Figures and Tables

**Table 1:** The table of sensitivity analysis, bias test, and meta-regression.

Outcome	Fixed model	Random model	Begg test (*P*)	Egger test (*P*)	Meta-regression (*P*)
HA	MD = −48.59, 95% CI: −51.00−46.18	MD = 57.15, 95% CI: −69.06−45.25	0.882	0.878	0.610; 0.007
LN	MD = −18.37, 95% CI: −19.69−17.00	MD = −35.04, 95% CI: −43.32−26.78	0.534	0.565	0.341; 0.033
PCIII	MD = −31.17, 95% CI: −33.68−28.68	MD = −29.32, 95% CI: −31.68−26.96	0.767	0.902	0.902; 0.071
IV-C	MD = −23.46, 95% CI: −24.91−22.00	MD = −38.98, 95% CI: −48.32−29.64	0.813	0.665	0.627; 0.303
AST	MD = −10.82, 95% CI: −12.10−9.55	MD = −9.92, 95% CI: −13.40−6.44	0.415	0.877	0.987; 0.866
ALT	MD = −15.69, 95% CI: −16.94−14.45	MD = −13.33, 95% CI: −16.99−9.68	0.608	0.153	0.496; 0.570
HBV-DNA	OR = 1.27, 95% CI: 0.92−1.76	OR = 1.33, 95% CI: 0.86−2.07	0.012	0.017	0.010; 0.722

## Data Availability

All the data in this study have been uploaded.

## Supplementary Materials

Supplementary Materials See Figures S1–S8 in the Supplementary Material for comprehensive image analysis, and see Tables S1–S8 for the raw data of the index in this study.
