# Supplementary material for: Clinical Efficacy and Safety of Eight Traditional Chinese Medicine Combined with Entecavir in the Treatment of Chronic Hepatitis B Liver Fibrosis in Adults: A Network Meta-Analysis
Source: Evid Based Complement Alternat Med. 2020 Sep 30;2020:7603410. doi: 10.1155/2020/7603410 (PMC7545414; doi:10.1155/2020/7603410)
Supplement: Supplementary Materials — See Figures S1–S8 in the Supplementary Material for comprehensive image analysis, and see Tables S1–S8 for the raw data of the index in this study. [file 7603410.f1.pdf]

Supplementary figure 1

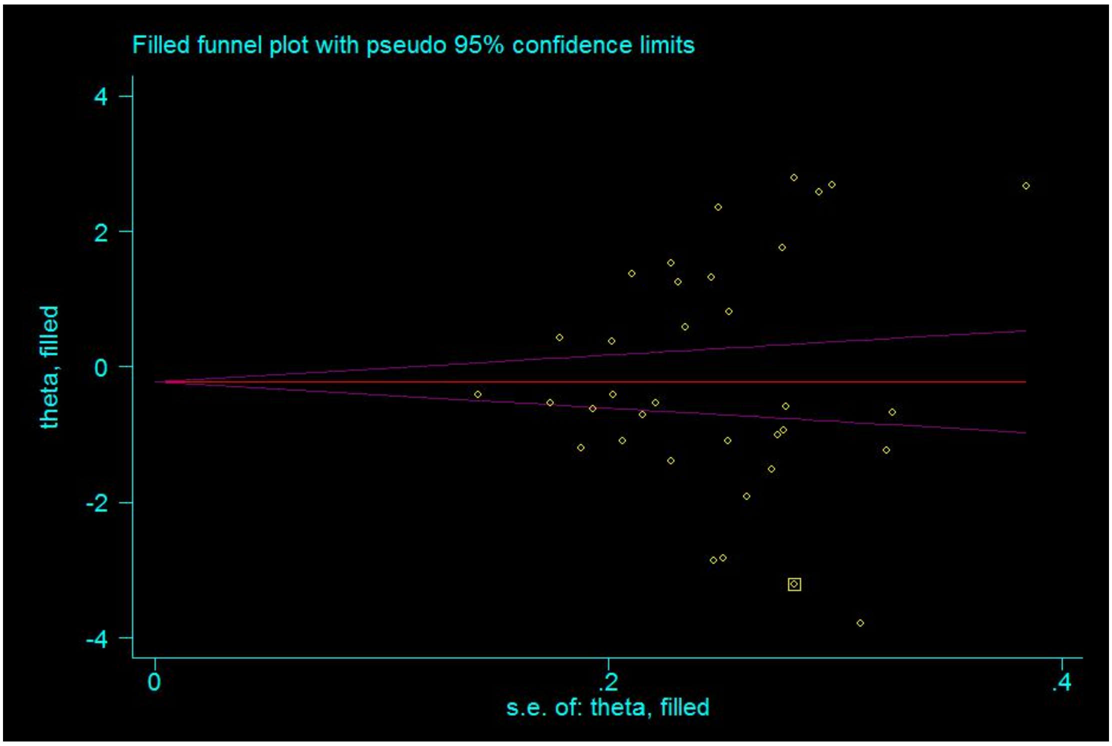

Supplementary figure 2

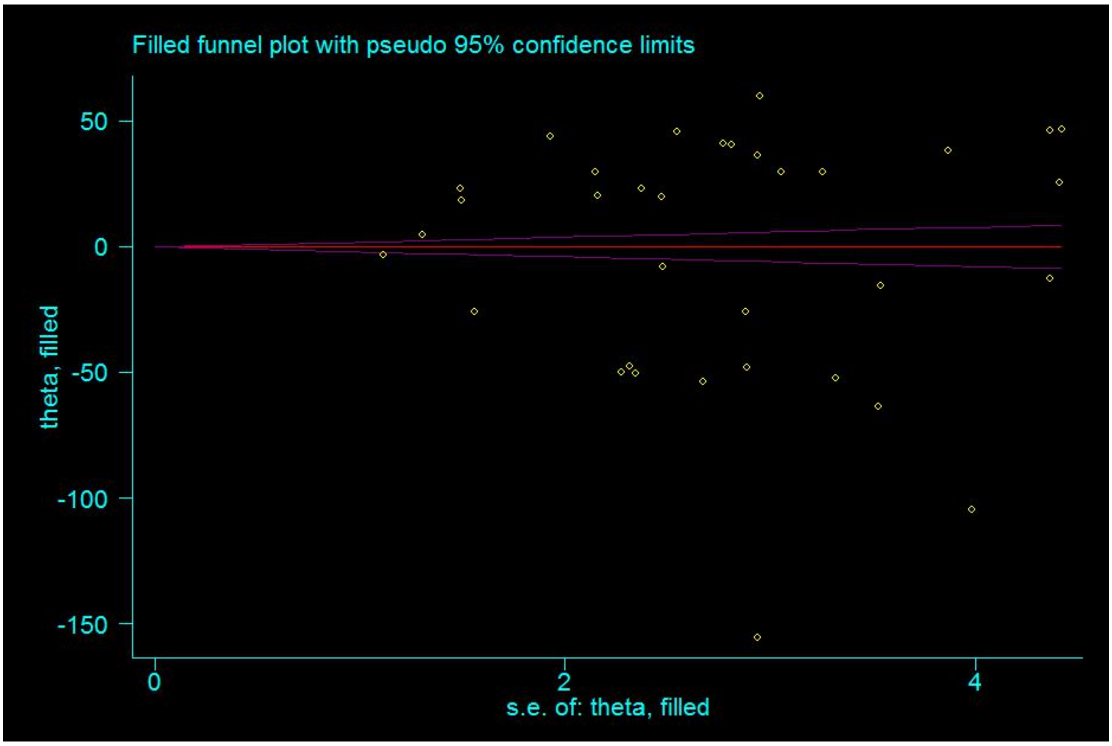

Supplementary figure 3

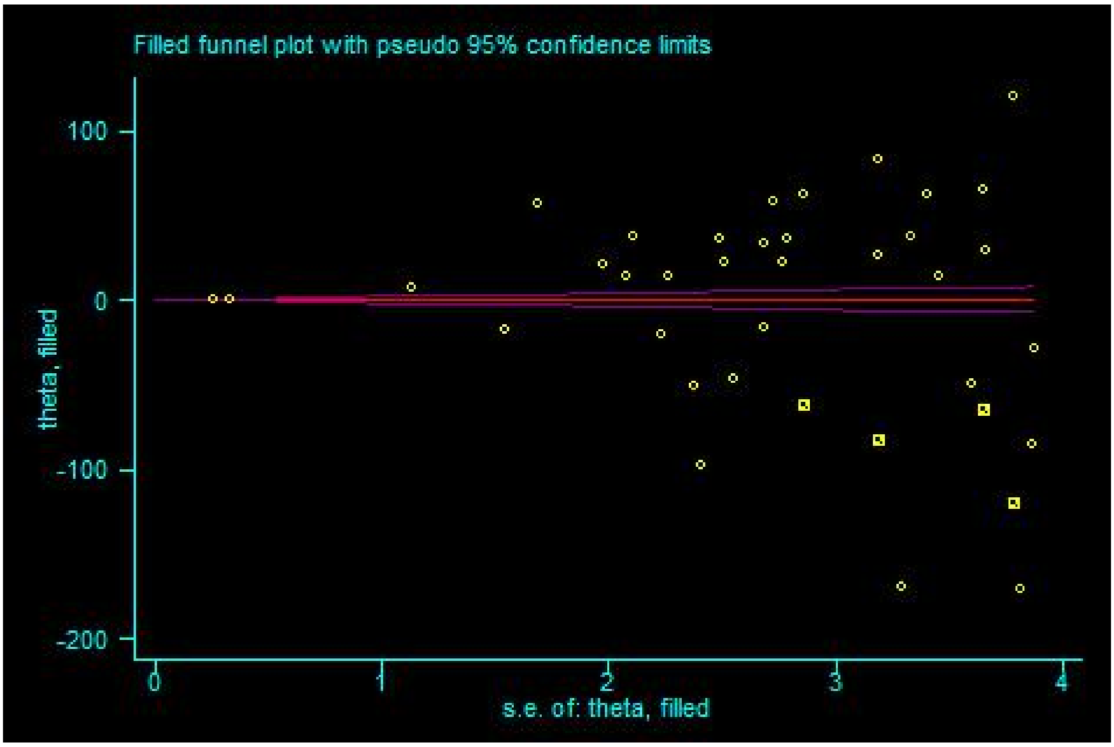

Supplementary figure 4

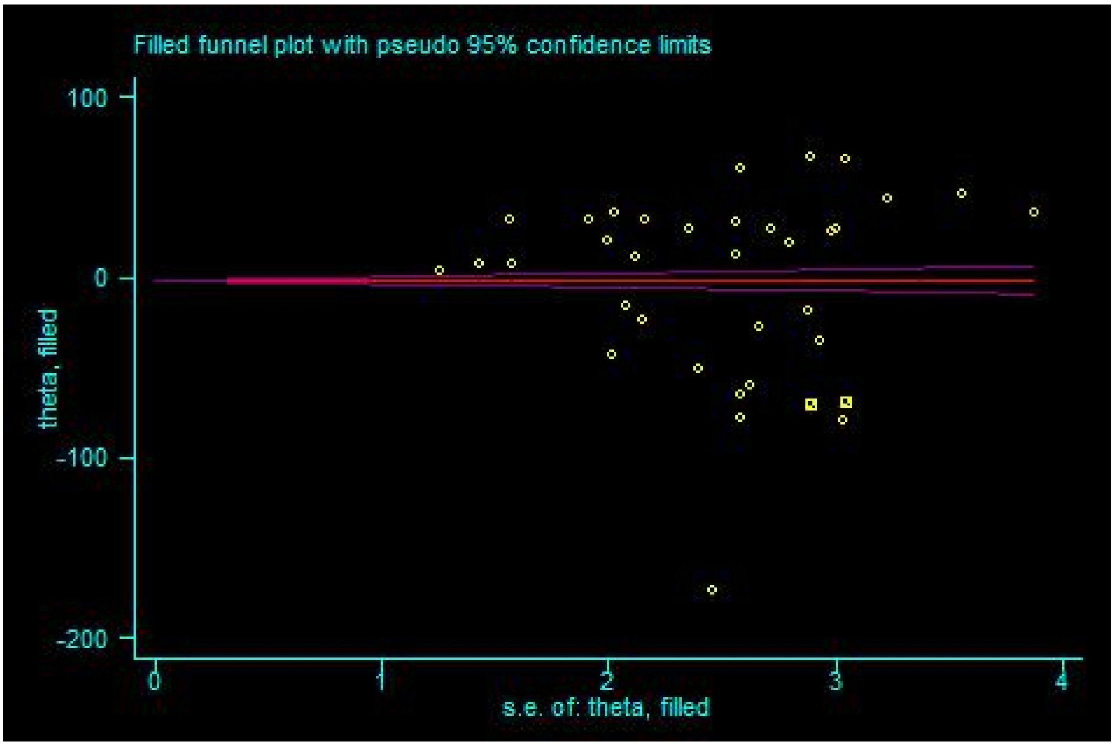

Supplementary figure 5

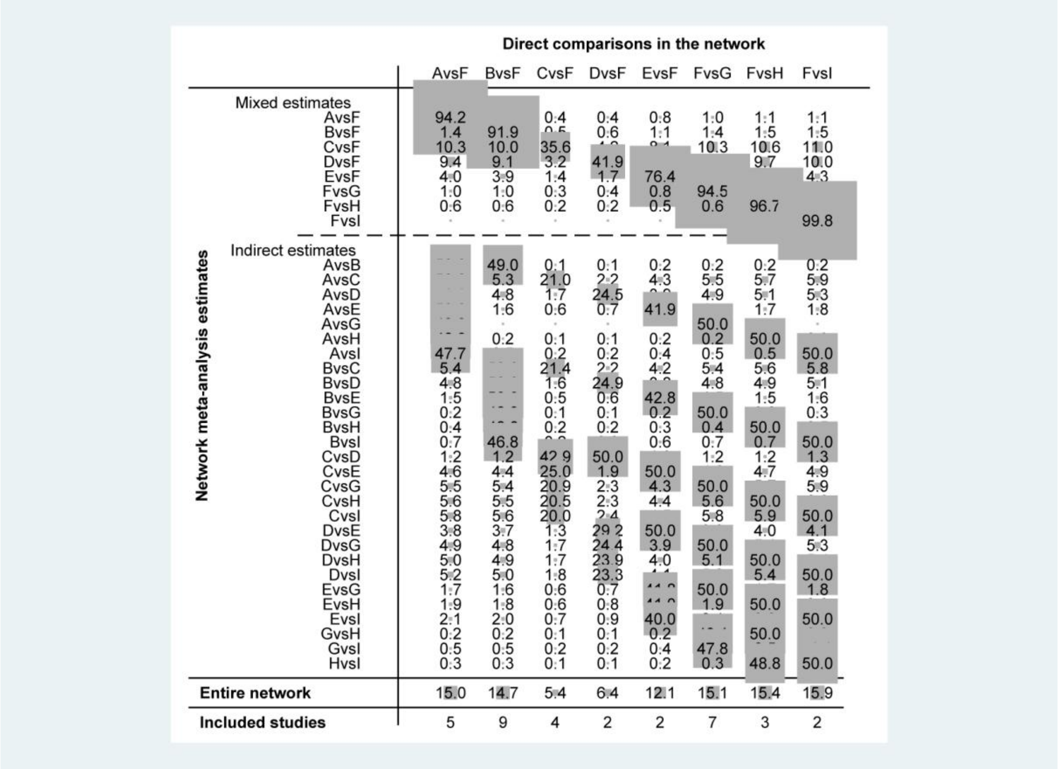

Direct comparisons in the network (rows: Network meta-analysis estimates)

	AvsF	BvsF	CvsF	DvsF	EvsF	FvsG	FvsH	FvsI
**Mixed estimates**								
AvsF	94.2	[illegible]	0.4	0.4	0.8	1.0	1.1	1.1
BvsF	1.4	91.9	[illegible]	0.6	1.1	1.4	1.5	1.5
CvsF	10.3	10.0	35.6	[illegible]	[illegible]	10.3	10.6	11.0
DvsF	9.4	9.1	3.2	41.9	[illegible]	[illegible]	9.7	10.0
EvsF	4.0	3.9	1.4	1.7	76.4	[illegible]	[illegible]	4.3
FvsG	1.0	1.0	0.3	0.4	0.8	94.5	[illegible]	[illegible]
FvsH	0.6	0.6	0.2	0.2	0.5	0.6	96.7	[illegible]
FvsI	.	.	.	.	.	[illegible]	[illegible]	99.8
**Indirect estimates**								
AvsB	[illegible]	49.0	0.1	0.1	0.2	0.2	0.2	0.2
AvsC	[illegible]	5.3	21.0	2.2	4.3	5.5	5.7	5.9
AvsD	[illegible]	4.8	1.7	24.5	[illegible]	4.9	5.1	5.3
AvsE	[illegible]	1.6	0.6	0.7	41.9	[illegible]	1.7	1.8
AvsG	[illegible]	.	.	.	[illegible]	50.0	[illegible]	.
AvsH	[illegible]	0.2	0.1	0.1	0.2	0.2	50.0	[illegible]
AvsI	47.7	[illegible]	0.2	0.2	0.4	0.5	0.5	50.0
BvsC	5.4	[illegible]	21.4	2.2	4.2	5.4	5.6	5.8
BvsD	4.8	[illegible]	1.6	24.9	[illegible]	4.8	4.9	5.1
BvsE	1.5	[illegible]	0.5	0.6	42.8	[illegible]	1.5	1.6
BvsG	0.2	[illegible]	0.1	0.1	0.2	50.0	[illegible]	0.3
BvsH	0.4	[illegible]	0.2	0.2	0.3	0.4	50.0	[illegible]
BvsI	0.7	46.8	[illegible]	[illegible]	0.6	0.7	0.7	50.0
CvsD	1.2	1.2	42.9	50.0	[illegible]	1.2	1.2	1.3
CvsE	4.6	4.4	25.0	1.9	50.0	[illegible]	4.7	4.9
CvsG	5.5	5.4	20.9	2.3	4.3	50.0	[illegible]	5.9
CvsH	5.6	5.5	20.5	2.3	4.4	5.6	50.0	[illegible]
CvsI	5.8	5.6	20.0	[illegible]	[illegible]	5.8	5.9	50.0
DvsE	3.8	3.7	1.3	29.2	50.0	[illegible]	4.0	4.1
DvsG	4.9	4.8	1.7	24.4	3.9	50.0	[illegible]	5.3
DvsH	5.0	4.9	1.7	23.9	4.0	5.1	50.0	[illegible]
DvsI	5.2	5.0	1.8	23.3	[illegible]	[illegible]	5.4	50.0
EvsG	1.7	1.6	0.6	0.7	[illegible]	50.0	[illegible]	1.8
EvsH	1.9	1.8	0.6	0.8	[illegible]	1.9	50.0	[illegible]
EvsI	2.1	2.0	0.7	0.9	40.0	[illegible]	[illegible]	50.0
GvsH	0.2	0.2	0.1	0.1	0.2	[illegible]	50.0	[illegible]
GvsI	0.5	0.5	0.2	0.2	0.4	47.8	[illegible]	[illegible]
HvsI	0.3	0.3	0.1	0.1	0.2	0.3	48.8	50.0
**Entire network**	15.0	14.7	5.4	6.4	12.1	15.1	15.4	15.9
**Included studies**	5	9	4	2	2	7	3	2

Supplementary figure 6

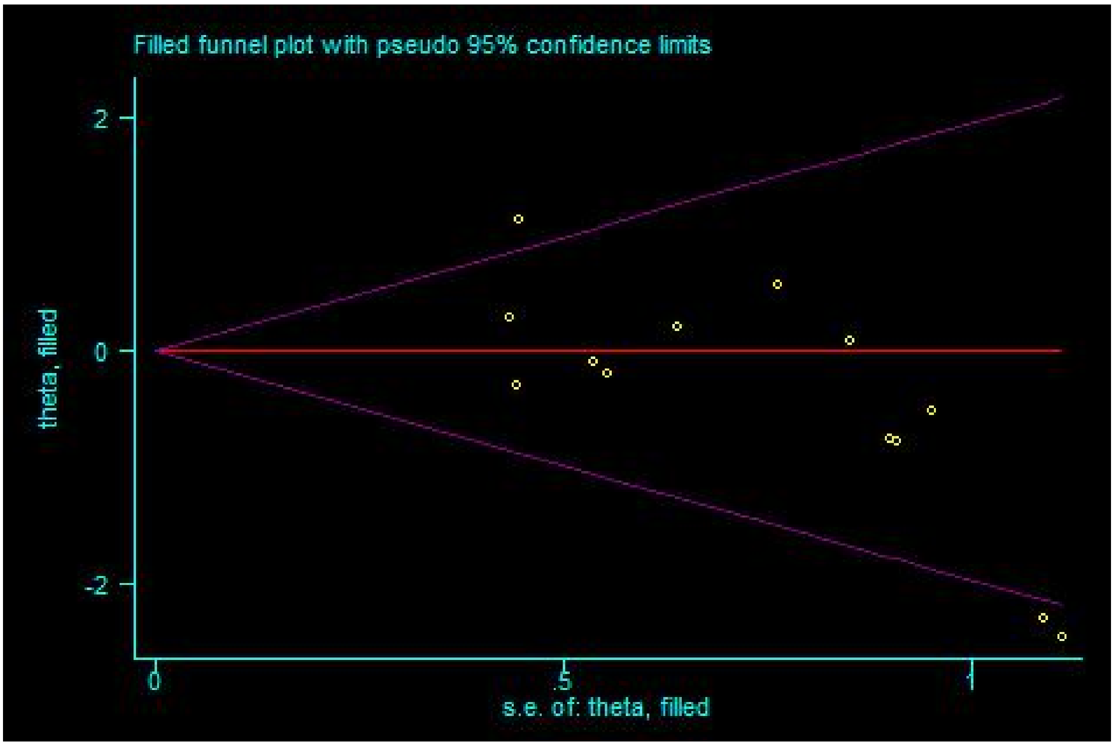

Supplementary figure 7

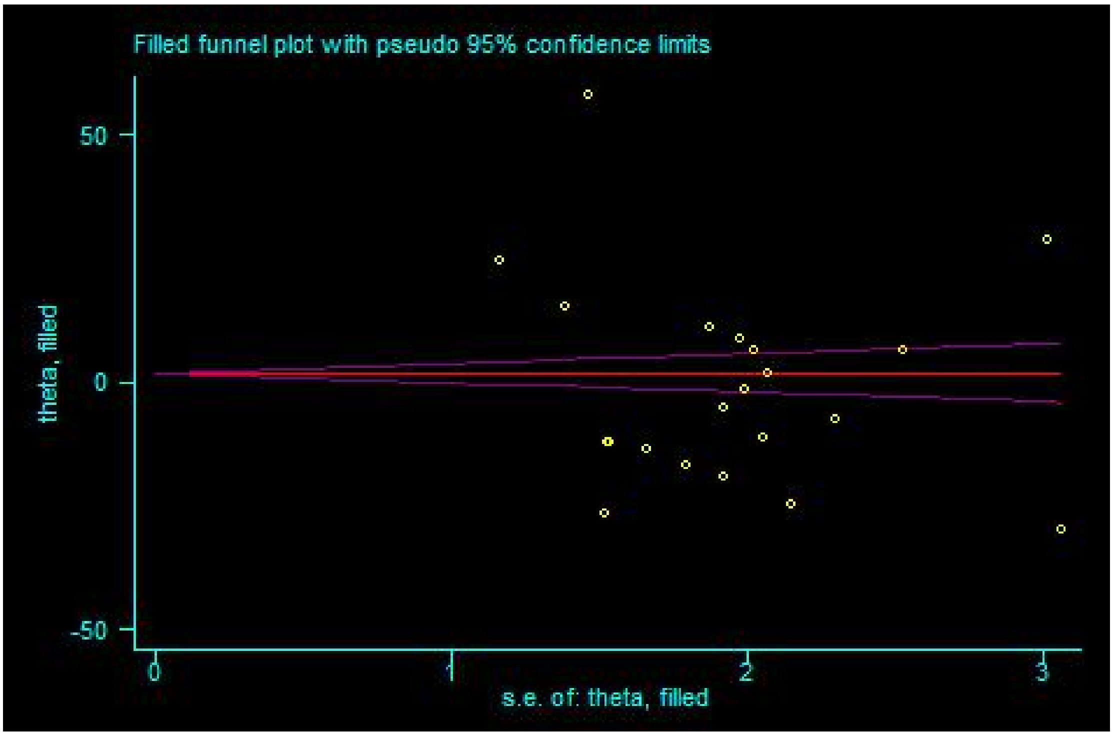

Supplementary figure 8

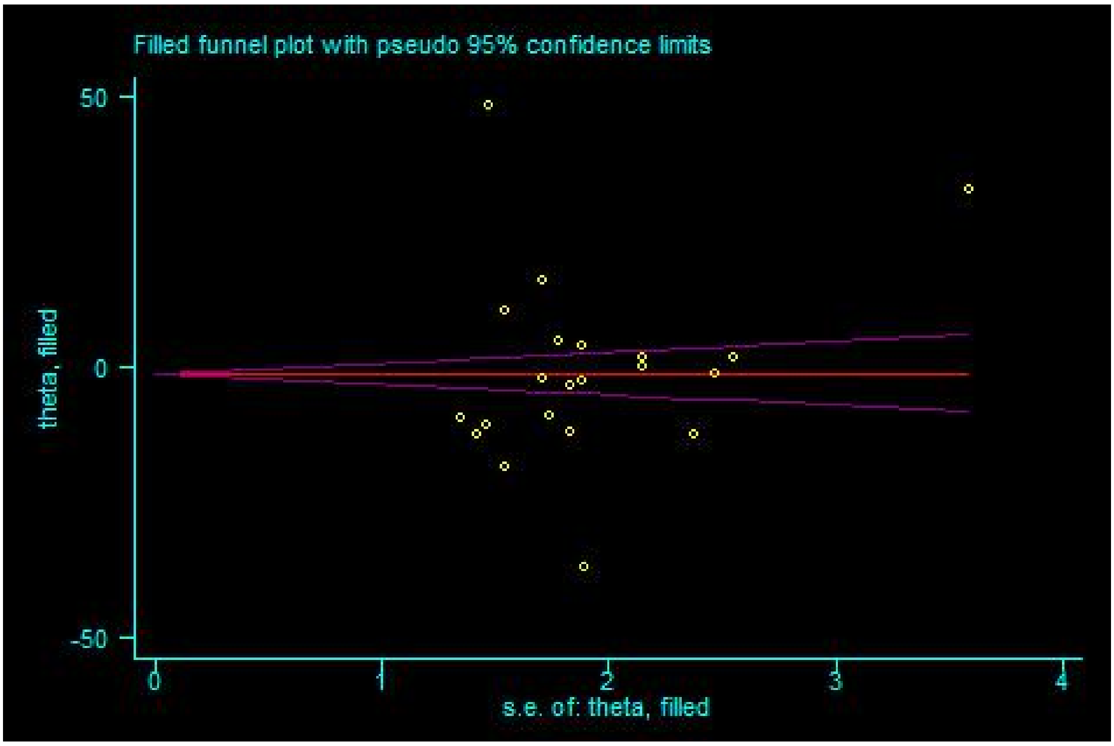

Supplementary table 1 Basic information

Study	Number of patients (C/E)	Age	Dosing method	Entecavir	TCM	Duration	Outcome
F Zhang 2017	35/35	43.8	Oral	0.5mg/Day	18g/Day (AHP)	24 weeks	HA, PCIII, IV-C, LN, ALT, AST, HBV-DNA
ZJ Huang 2016	66/68	42.1	Oral	0.5mg/Day	12g/Day (AHP)	48 weeks	ALT, AST, HA, PCIII, IV-C, LN, HBV-DNA
W Guo 2010	42/43	36.5	Oral	0.5mg/Day	18g/Day (AHP)	48 weeks	HBVDNA, HA, PCIII, IV-C, LN
YH Zeng 2013	30/30	40.2	Oral	0.5mg/Day	12g/Day (AHP)	48 weeks	ALT, AST, HA, PCIII, IV-C, LN
HD Qi 2008	28/30	NA	Oral	0.5mg/Day	12g/Day (AHP)	48 weeks	HA, PCIII, IV-C, LN, HBV-DNA
CR Shen 2010	50/46	44.8	Oral	0.5mg/Day	6g/Day (BRT)	48 weeks	ALT, AST, HA, PCIII, IV-C, LN
DJ Chen 2017	36/36	45.9	Oral	0.5mg/Day	18g/Day (BRT)	48 weeks	HA, PCIII, IV-C, LN
HT Zhang 2015	100/100	NA	Oral	0.5mg/Day	6g/Day (BRT)	48 weeks	ALT, AST, HA, PCIII, IV-C, LN
GZ Wu 2017	47/45	43.5	Oral	0.5mg/Day	12 capsules/Day (BRT)	48 weeks	ALT, AST, HBV-DNA, HA, PCIII, IV-C, LN
ZG Zhang 2012	43/43	44	Oral	0.5mg/Day	12 capsules/Day (BRT)	48 weeks	HA, PCIII, IV-C, LN
L Xiao 2013	54/54	45.5	Oral	0.5mg/Day	12 capsules/Day (BRT)	48 weeks	HA, PCIII, IV-C, LN, ALT, AST
YY Sun 2017	38/38	42.2	Oral	0.5mg/Day	60mg/Day (BRT)	48 weeks	HBV-DNA, HA, PCIII, IV-C, LN
XL Zhang 2016	63/71	56.5	Oral	0.5mg/Day	60mg/Day (BRT)	48 weeks	HA, PCIII, IV-C, LN
RF Zhang 2014	55/57	NA	Oral	0.5mg/Day	6g/Day (BRT)	48 weeks	ALT, AST, HA, PCIII, IV-C, LN, HBV-DNA
Q Yang 2015	50/50	45.1	Oral	0.5mg/Day	4.5g/Day (LWT)	48 weeks	ALT, AST, HA, PCIII, IV-C, LN
YQ Zhao 2015	55/57	43.5	Oral	0.5mg/Day	4.5g/Day (LWT)	48 weeks	ALT, AST, HA, PCIII, IV-C, LN
GQ Chen 2016	45/45	42	Oral	0.5mg/Day	4.5g/Day (LWT)	48 weeks	HA, PCIII, IV-C, LN, HBV-DNA, ALT, AST
FL Long 2011	59/57	32.8	Injection	0.5mg/Day	10ml/Day (DI)	4 weeks	HA, PCIII, IV-C, LN, ALT, AST
XQ Chen 2016	68/68	45.2	Injection	0.5mg/Day	10ml/Day (DI)	12 weeks	HA, PCIII, IV-C, LN
Y Zuo 2015	43/43	40.9	Oral	0.5mg/Day	30capsules/Day(DDT)	48 weeks	HA, PCIII, IV-C, LN
JH Zhang 2014	23/23	42.5	Oral	0.5mg/Day	750mg/Day (DDT)	24 weeks	HA, PCIII, IV-C, LN, HBV-DNA
YJ Qiu 2017	64/64	48.3	Oral	0.5mg/Day	30capsules/Day(DDT)	24 weeks	HA, PCIII, IV-C, LN
YL Fan 2009	30/30	43.3	Oral	0.5mg/Day	750mg/Day (DDT)	24 weeks	HA, PCIII, IV-C, LN, HBV-DNA

RQ Fan 2013	35/38	46.3	Oral	0.5mg/Day	4.5g/Day (FHC)	48 weeks	ALT, AST, HA, PCIII, IV-C, LN, HBV-DNA
LL Wu 2013	55/55	48.1	Oral	0.5mg/Day	4.5g/Day (FHC)	48 weeks	ALT, AST, HA, PCIII, IV-C, LN
HS Xie 2016	63/65	38.4	Oral	0.5mg/Day	4.5g/Day (FHC)	48 weeks	ALT, AST, HA, PCIII, IV-C, LN
YZ Song 2012	32/46	38.5	Oral	0.5mg/Day	4.5g/Day (FHC)	48 weeks	HA, PCIII, IV-C, LN
SQ Pang 2013	42/43	36.5	Oral	0.5mg/Day	4.5g/Day (FHC)	48 weeks	ALT, AST, HA, PCIII, IV-C, LN
ZF Shangguan 2013	40/40	47.5	Oral	0.5mg/Day	4.5g/Day (FHC)	48 weeks	ALT, AST, HA, PCIII, IV-C, LN
YH Gong 2010	24/28	46	Oral	0.5mg/Day	15 capsules/Day (FHC)	48 weeks	ALT, AST, HA, PCIII, IV-C, LN
XP Pan 2016	50/50	60.8	Oral	0.5mg/Day	3g/Day (TSC)	48 weeks	ALT, AST, HA, PCIII, IV-C, LN
ZH Hu 2016	43/43	45.9	Oral	0.5mg/Day	3g/Day (TSC)	48 weeks	ALT, AST, HA, PCIII, IV-C, LN, HBV-DNA
C Xie 2016	50/50	39.6	Oral	0.5mg/Day	8 capsules/Day (DZC)	48 weeks	ALT, AST, HA, PCIII, IV-C, LN, HBV-DNA
J Shi 2013	20/20	42.4	Oral	0.5mg/Day	8 capsules/Day (DZC)	48 weeks	ALT, AST, HA, PCIII, IV-C, LN, HBV-DNA

Supplementary table 2 HA

**Entecavir**	**TSC+E**	**LWT+E**	**FHC+E**	**DZC+E**	**DI+E**	**DDP+E**	**BRT+E**	**AHP+E**
Entecavir	-91.54 (-152.79,-30.29)	-30.08 (-80.08,19.92)	-61.97 (-94.74,-29.21)	-33.70 (-95.09,27.69)	-62.12 (-123.34,-0.89)	-24.29 (-67.62,19.04)	-45.03 (-73.92,-16.14)	-69.20 (-108.02,-30.37)
91.54 (30.29,152.79)	**TSC+E**	61.46 (-17.61,140.53)	29.57 (-39.90,99.03)	57.84 (-28.88,144.56)	29.42 (-57.18,116.02)	67.25 (-7.78,142.28)	46.51 (-21.21,114.23)	22.34 (-50.17,94.86)
30.08 (-19.92,80.08)	-61.46 (-140.53,17.61)	**LWT+E**	-31.89 (-91.67,27.89)	-3.62 (-82.79,75.56)	-32.04 (-111.08,47.01)	5.79 (-60.37,71.96)	-14.95 (-72.69,42.80)	-39.11 (-102.42,24.19)
61.97 (29.21,94.74)	-29.57 (-99.03,39.90)	31.89 (-27.89,91.67)	**FHC+E**	28.27 (-41.31,97.86)	-0.14 (-69.58,69.29)	37.68 (-16.64,92.01)	16.95 (-26.74,60.63)	-7.22 (-58.02,43.58)
33.70 (-27.69,95.09)	-57.84 (-144.56,28.88)	3.62 (-75.56,82.79)	-28.27 (-97.86,41.31)	**DZC+E**	-28.42 (-115.12,58.28)	9.41 (-65.73,84.55)	-11.33 (-79.17,56.52)	-35.49 (-108.13,37.14)
62.12 (0.89,123.34)	-29.42 (-116.02,57.18)	32.04 (-47.01,111.08)	0.14 (-69.29,69.58)	28.42 (-58.28,115.12)	**DI+E**	37.83 (-37.18,112.83)	17.09 (-50.61,84.79)	-7.08 (-79.57,65.42)
24.29 (-19.04,67.62)	-67.25 (-142.28,7.78)	-5.79 (-71.96,60.37)	-37.68 (-92.01,16.64)	-9.41 (-84.55,65.73)	-37.83 (-112.83,37.18)	**DDP+E**	-20.74 (-72.82,31.34)	-44.91 (-103.08,13.27)
45.03 (16.14,73.92)	-46.51 (-114.23,21.21)	14.95 (-42.80,72.69)	-16.95 (-60.63,26.74)	11.33 (-56.52,79.17)	-17.09 (-84.79,50.61)	20.74 (-31.34,72.82)	**BRT+E**	-24.17 (-72.56,24.22)
69.20 (30.37,108.02)	-22.34 (-94.86,50.17)	39.11 (-24.19,102.42)	7.22 (-43.58,58.02)	35.49 (-37.14,108.13)	7.08 (-65.42,79.57)	44.91 (-13.27,103.08)	24.17 (-24.22,72.56)	**AHP+E**

Supplementary table 3 LN

Entecavir	TSC+E	LWT+E	FHC+E	DZC+E	DI+E	DDP+E	BRT+E	AHP+E
**Entecavir**	-48.99	-78.42	-45.29	3.86	-19.39	-45.71	-21.71	-28.84
	(-91.65,-6.33)	(-113.26,-43.58)	(-68.14,-22.43)	(-38.91,46.63)	(-62.00,23.23)	(-75.93,-15.49)	(-41.85,-1.56)	(-55.90,-1.77)
48.99 (6.33,91.65)	**TSC+E**	-29.43	3.70	52.85	29.60	3.28	27.28	20.16
		(-84.51,25.65)	(-44.69,52.10)	(-7.56,113.25)	(-30.69,89.90)	(-48.99,55.56)	(-19.89,74.46)	(-30.36,70.67)
78.42 (43.58,113.26)	29.43	**LWT+E**	33.13	82.28	59.03	32.71	56.71	49.58
	(-25.65,84.51)		(-8.54,74.80)	(27.11,137.44)	(3.98,114.08)	(-13.41,78.83)	(16.47,96.96)	(5.47,93.70)
45.29 (22.43,68.14)	-3.70	-33.13	**FHC+E**	49.14	25.90	-0.42	23.58	16.45
	(-52.10,44.69)	(-74.80,8.54)		(0.65,97.64)	(-22.46,74.26)	(-38.31,37.47)	(-6.88,54.05)	(-18.97,51.88)
-3.86	-52.85	-82.28	-49.14	**DZC+E**	-23.24	-49.56	-25.56	-32.69
(-46.63,38.91)	(-113.25,7.56)	(-137.44,-27.11)	(-97.64,-0.65)		(-83.62,37.13)	(-101.93,2.80)	(-72.84,21.71)	(-83.30,17.92)
19.39	-29.60	-59.03	-25.90	23.24	**DI+E**	-26.32	-2.32	-9.45
(-23.23,62.00)	(-89.90,30.69)	(-114.08,-3.98)	(-74.26,22.46)	(-37.13,83.62)		(-78.56,25.92)	(-49.45,44.82)	(-59.93,41.04)
45.71 (15.49,75.93)	-3.28	-32.71	0.42	49.56	26.32	**DDP+E**	24.00	16.87
	(-55.56,48.99)	(-78.83,13.41)	(-37.47,38.31)	(-2.80,101.93)	(-25.92,78.56)		(-12.31,60.32)	(-23.69,57.44)
21.71 (1.56,41.85)	-27.28	-56.71	-23.58	25.56	2.32	-24.00	**BRT+E**	-7.13
	(-74.46,19.89)	(-96.96,-16.47)	(-54.05,6.88)	(-21.71,72.84)	(-44.82,49.45)	(-60.32,12.31)		(-40.86,26.61)
28.84 (1.77,55.90)	-20.16	-49.58	-16.45	32.69	9.45	-16.87	7.13	**AHP+E**
	(-70.67,30.36)	(-93.70,-5.47)	(-51.88,18.97)	(-17.92,83.30)	(-41.04,59.93)	(-57.44,23.69)	(-26.61,40.86)	

Supplementary table 4 PCIII

Entecavir	TSC+E	LWT+E	FHC+E	DZC+E	DI+E	DDP+E	BRT+E	AHP+E
**Entecavir**	-48.13	-29.41	-76.65	-11.11	-27.40	-53.89	-36.46	-43.70
	(-108.95,12.70)	(-79.08,20.26)	(-109.21,-44.09)	(-71.88,49.67)	(-88.22,33.41)	(-96.92,-10.86)	(-65.14,-7.78)	(-82.21,-5.19)
48.13	**TSC+E**	18.72	-28.52	37.02	20.72	-5.76	11.67	4.43
(-12.70,108.95)		(-59.81,97.24)	(-97.52,40.47)	(-48.97,123.00)	(-65.30,106.74)	(-80.27,68.75)	(-55.58,78.92)	(-67.57,76.42)
29.41	-18.72	**LWT+E**	-47.24	18.30	2.00	-24.48	-7.05	-14.29
(-20.26,79.08)	(-97.24,59.81)		(-106.63,12.15)	(-60.19,96.79)	(-76.52,80.53)	(-90.19,41.24)	(-64.40,50.30)	(-77.14,48.56)
76.65	28.52	47.24	**FHC+E**	65.54	49.25	22.76	40.19	32.95
(44.09,109.21)	(-40.47,97.52)	(-12.15,106.63)		(-3.40,134.49)	(-19.74,118.23)	(-31.20,76.72)	(-3.20,83.58)	(-17.48,83.38)
11.11	-37.02	-18.30	-65.54	**DZC+E**	-16.30	-42.78	-25.35	-32.59
(-49.67,71.88)	(-123.00,48.97)	(-96.79,60.19)	(-134.49,3.40)		(-102.28,69.68)	(-117.25,31.69)	(-92.55,41.85)	(-104.54,39.36)
27.40	-20.72	-2.00	-49.25	16.30	**DI+E**	-26.48	-9.05	-16.29
(-33.41,88.22)	(-106.74,65.30)	(-80.53,76.52)	(-118.23,19.74)	(-69.68,102.28)		(-100.99,48.02)	(-76.30,58.19)	(-88.28,55.69)
53.89	5.76	24.48	-22.76	42.78	26.48	**DDP_E**	17.43	10.19
(10.86,96.92)	(-68.75,80.27)	(-41.24,90.19)	(-76.72,31.20)	(-31.69,117.25)	(-48.02,100.99)		(-34.28,69.14)	(-47.56,67.93)
36.46	-11.67	7.05	-40.19	25.35	9.05	-17.43	**BRT+E**	-7.24
(7.78,65.14)	(-78.92,55.58)	(-50.30,64.40)	(-83.58,3.20)	(-41.85,92.55)	(-58.19,76.30)	(-69.14,34.28)		(-55.26,40.77)
43.70	-4.43	14.29	-32.95	32.59	16.29	-10.19	7.24	**AHP+E**
(5.19,82.21)	(-76.42,67.57)	(-48.56,77.14)	(-83.38,17.48)	(-39.36,104.54)	(-55.69,88.28)	(-67.93,47.56)	(-40.77,55.26)	

Supplementary table 5 IV-C

Entecavir	TSC+E	LWT+E	FHC+E	DZC+E	DI+E	DDP+E	BRT+E	AHP+E
**Entecavir**	-19.54	-93.52	-49.92	-7.93	-20.13	-36.04	-37.95	-24.83
	(-55.97,16.88)	(-123.28,-63.75)	(-69.43,-30.40)	(-44.34,28.48)	(-56.50,16.23)	(-61.83,-10.24)	(-55.16,-20.74)	(-47.92,-1.75)
19.54	**TSC+E**	-73.97	-30.37	11.61	-0.59	-16.49	-18.40	-5.29
(-16.88,55.97)		(-121.01,-26.93)	(-71.69,10.95)		(-52.06,50.88)	(-61.12,28.14)	(-58.69,21.88)	(-48.41,37.83)
				(-39.89,63.12)				
93.52	73.97	**LWT+E**	43.60	85.59	73.38	57.48	55.57	68.68
(63.75,123.28)	(26.93,121.01)		(8.01,79.19)	(38.56,132.62)	(26.39,120.38)	(18.09,96.87)	(21.19,89.95)	(31.01,106.35)
49.92	30.37	-43.60	**FHC+E**	41.99	29.79	13.88	11.97 (-14.05,37.99)	25.08
(30.40,69.43)	(-10.95,71.69)	(-79.19,-8.01)		(0.67,83.30)	(-11.48,71.06)	(-18.46,46.22)		(-5.14,55.31)
7.93	-11.61	-85.59	-41.99	**DZC+E**	-12.20	-28.11	-30.02	-16.90
(-28.48,44.34)	(-63.12,39.89)	(-132.62,-38.56)	(-83.30,-0.67)		(-63.67,39.26)	(-72.73,16.52)	(-70.29,10.26)	(-60.02,26.21)
20.13	0.59	-73.38	-29.79	12.20	**DI+E**	-15.90	-17.81	-4.70
(-16.23,56.50)	(-50.88,52.06)	(-120.38,-26.39)	(-71.06,11.48)	(-39.26,63.67)		(-60.49,28.68)	(-58.05,22.42)	(-47.78,38.37)
36.04	16.49	-57.48	-13.88	28.11	15.90	**DDP+E**	-1.91	11.20
(10.24,61.83)	(-28.14,61.12)	(-96.87,-18.09)	(-46.22,18.46)	(-16.52,72.73)	(-28.68,60.49)		(-32.92,29.10)	(-23.41,45.82)
37.95	18.40	-55.57	-11.97	30.02	17.81	1.91	**BRT+E**	13.11
(20.74,55.16)	(-21.88,58.69)	(-89.95,-21.19)	(-37.99,14.05)	(-10.26,70.29)	(-22.42,58.05)	(-29.10,32.92)		(-15.68,41.91)
24.83	5.29	-68.68	-25.08	16.90	4.70	-11.20	-13.11	**AHP+E**
(1.75,47.92)	(-37.83,48.41)	(-106.35,-31.01)	(-55.31,5.14)	(-26.21,60.02)	(-38.37,47.78)	(-45.82,23.41)	(-41.91,15.68)	

Supplementary table 6 ALT

Entecavir	TSC+E	LWT+E	FHC+E	DZC+E	DI+E	BRT+E	AHP+E
**Entecavir**	-12.87 (-23.65,-2.09)	-20.20 (-29.18,-11.22)	-15.48 (-21.77,-9.19)	-6.28 (-21.71,9.15)	-24.80 (-39.89,-9.71)	-5.23 (-12.14,1.69)	-17.02 (-25.94,-8.09)
12.87 (2.09,23.65)	**TSC+E**	-7.33 (-21.35,6.70)	-2.60 (-15.08,9.87)	6.59 (-12.23,25.42)	-11.93 (-30.47,6.62)	7.65 (-5.16,20.45)	-4.15 (-18.14,9.85)
20.20 (11.22,29.18)	7.33 (-6.70,21.35)	**LWT+E**	4.72 (-6.24,15.69)	13.92 (-3.94,31.78)	-4.60 (-22.16,12.96)	14.97 (3.64,26.31)	3.18 (-9.47,15.83)
15.48 (9.19,21.77)	2.60 (-9.87,15.08)	-4.72 (-15.69,6.24)	**FHC+E**	9.20 (-7.47,25.86)	-9.32 (-25.67,7.02)	10.25 (0.90,19.60)	-1.54 (-12.46,9.38)
6.28 (-9.15,21.71)	-6.59 (-25.42,12.23)	-13.92 (-31.78,3.94)	-9.20 (-25.86,7.47)	**DZC+E**	-18.52 (-40.11,3.07)	1.05 (-15.86,17.97)	-10.74 (-28.57,7.09)
24.80 (9.71,39.89)	11.93 (-6.62,30.47)	4.60 (-12.96,22.16)	9.32 (-7.02,25.67)	18.52 (-3.07,40.11)	**DI+E**	19.57 (2.97,36.18)	7.78 (-9.75,25.31)
5.23 (-1.69,12.14)	-7.65 (-20.45,5.16)	-14.97 (-26.31,-3.64)	-10.25 (-19.60,-0.90)	-1.05 (-17.97,15.86)	-19.57 (-36.18,-2.97)	**BRT+E**	-11.79 (-23.09,-0.50)
17.02 (8.09,25.94)	4.15 (-9.85,18.14)	-3.18 (-15.83,9.47)	1.54 (-9.38,12.46)	10.74 (-7.09,28.57)	-7.78 (-25.31,9.75)	11.79 (0.50,23.09)	**AHP+E**

Supplementary table 7 AST

**Entecavir**	**TSC+E**	**LWT+E**	**FHC+E**	**DZC+E**	**DI+E**	**BRT+E**	**AHP+E**
**Entecavir**	-11.31 (-23.45,0.83)	-14.24 (-24.26,-4.21)	-10.39 (-17.46,-3.31)	1.77 (-15.50,19.04)	-10.30 (-27.51,6.91)	-2.59 (-10.39,5.20)	-22.18 (-32.33,-12.03)
11.31 (-0.83,23.45)	**TSC+E**	-2.92 (-18.67,12.82)	0.93 (-13.13,14.98)	13.08 (-8.03,34.19)	1.01 (-20.05,22.07)	8.72 (-5.72,23.15)	-10.87 (-26.69,4.96)
14.24 (4.21,24.26)	2.92 (-12.82,18.67)	**LWT+E**	3.85 (-8.42,16.12)	16.01 (-3.96,35.97)	3.94 (-15.98,23.85)	11.64 (-1.06,24.34)	-7.94 (-22.21,6.32)
10.39 (3.31,17.46)	-0.93 (-14.98,13.13)	-3.85 (-16.12,8.42)	**FHC+E**	12.16 (-6.51,30.82)	0.09 (-18.52,18.69)	7.79 (-2.74,18.32)	-11.79 (-24.17,0.58)
-1.77 (-19.04,15.50)	-13.08 (-34.19,8.03)	-16.01 (-35.97,3.96)	-12.16 (-30.82,6.51)	**DZC+E**	-12.07 (-36.45,12.31)	-4.36 (-23.31,14.58)	-23.95 (-43.98,-3.91)
10.30 (-6.91,27.51)	-1.01 (-22.07,20.05)	-3.94 (-23.85,15.98)	-0.09 (-18.69,18.52)	12.07 (-12.31,36.45)	**DI+E**	7.71 (-11.19,26.60)	-11.88 (-31.86,8.10)
2.59 (-5.20,10.39)	-8.72 (-23.15,5.72)	-11.64 (-24.34,1.06)	-7.79 (-18.32,2.74)	4.36 (-14.58,23.31)	-7.71 (-26.60,11.19)	**BRT+E**	-19.58 (-32.38,-6.78)
22.18 (12.03,32.33)	10.87 (-4.96,26.69)	7.94 (-6.32,22.21)	11.79 (-0.58,24.17)	23.95 (3.91,43.98)	11.88 (-8.10,31.86)	19.58 (6.78,32.38)	**AHP+E**

Supplementary table 8 Adverse events

Study	Number of patients(C/E)	Adverse reactions in the control group	Adverse reactions in the experiment group
F Zhang 2017	35/35	Increased frequency of defecation (2)	No adverse reaction
GZ Wu 2017	47/45	Discomfort in the stomach (4)	Discomfort in the stomach (5)
YY Sun 2017	38/38	Nausea (1); dizziness (1)	Nausea (1); dizziness (1); fatigue (1)
XL Zhang 2016	83/71	Gastrointestinal symptoms	Gastrointestinal symptoms, vertigo and fatigue
RF Zhang 2014	55/57	Discomfort in the stomach (5)	Discomfort in the stomach (4)
GQ Chen 2016	45/45	Increased serum CK	No adverse reaction
XQ Chen 2016	68/68	Nausea(3); allergy (1)	Nausea(2)
YJ Qiu 2017	64/64	Nausea (3); bloating (3);loss of appetite (2)	Nausea (2); bloating (1); loss of appetite (1)
RQ Fan 2013	35/38	No adverse reaction	Discomfort in the stomach (2)
